# Reversible chorea secondary to uremia in an older adult

**DOI:** 10.1002/agm2.12062

**Published:** 2019-05-06

**Authors:** Abhijith Rajaram Rao, Pramod Kumar, Venugopalan Gunasekaran, Aparajit Ballav Dey

**Affiliations:** ^1^ Department of Geriatric Medicine All India Institute of Medical Sciences New Delhi India

**Keywords:** chorea, older adult, uremia

## 1 INTRODUCTION

Chorea is characterized by the continuous flow of random brief involuntary muscle contractions. The movements can involve any body part, including the face, limbs, trunk, or neck. The unpredictable nature of chorea is a feature that distinguishes it from tremor and dystonia. It can be due to multiple causes. Here, we report a case of chorea that was secondary to uremia.

## 2 CASE REPORT

A 78‐year‐old woman with a past history of hypertension, diabetes, and chronic kidney disease (CKD) stage 4 presented to us with altered sensorium of 45 days' duration. It was acute in onset with a fluctuating course associated with incoherent speech and decreased oral intake. Ten days prior to admission she had developed involuntary and continuous abnormal movements of both her upper limbs. Her medications included metoprolol, aspirin, atorvastatin, linagliptin, and pantoprazole.

**Figure 1:**
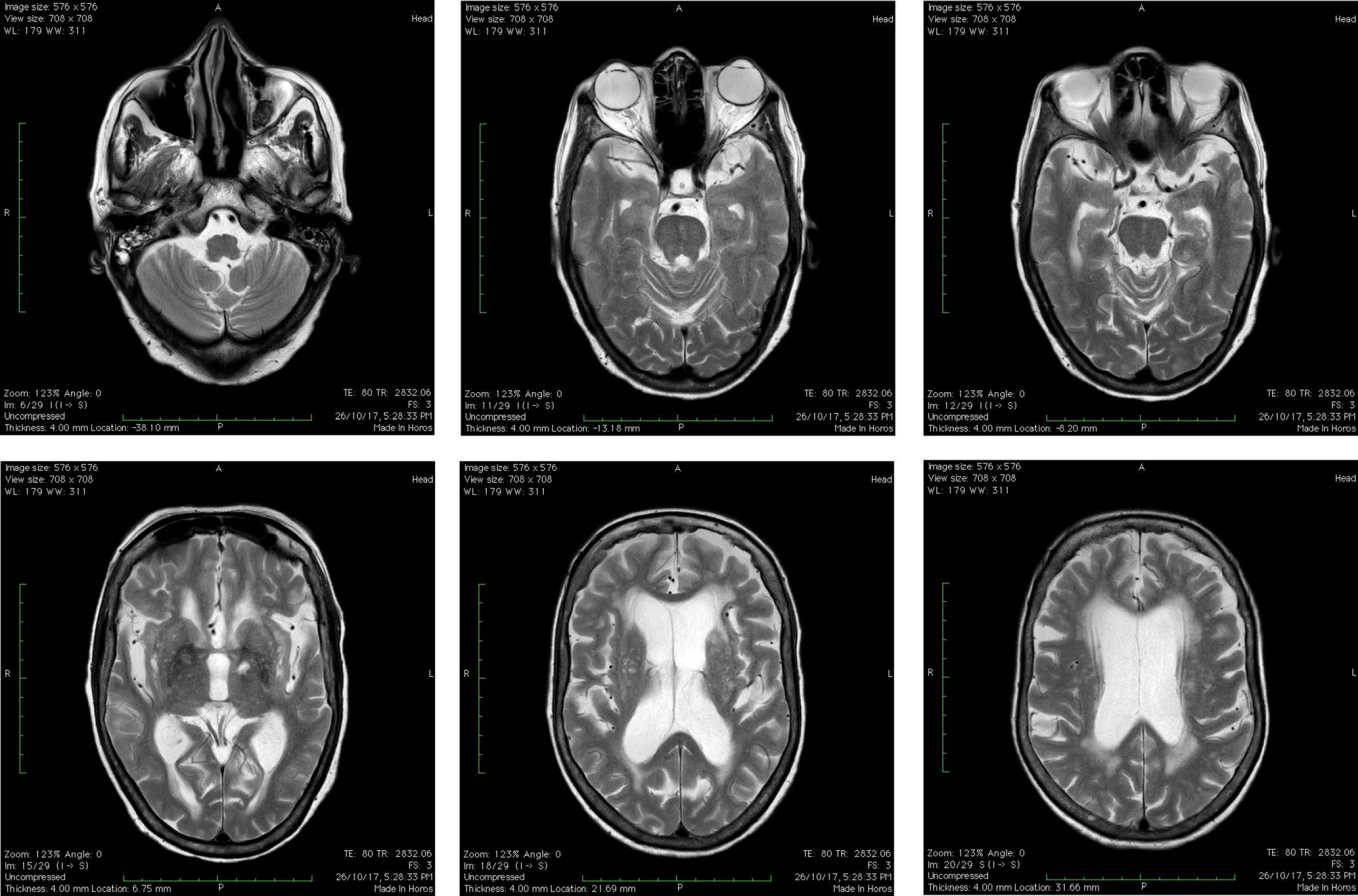
Magnetic resonance imaging of the patient's brain showing normal age‐related changes only

On examination, the patient was drowsy with a Glasgow Coma Scale score of 5/15 (Eye, 3; Verbal, 1; Motor, 1). Vitals were stable with normal blood sugar level. Involuntary movements were present in bilateral upper limbs (left > right) and were high amplitude, proximal, non‐rhythmic, present at rest and subsided while asleep, with increased tone in all four limbs. Laboratory testing revealed blood urea of 227 mg/dL (10‐40 mg/dL), serum creatinine of 3.5 mg/dL (0.5‐1.0 mg/dL), pyuria (50‐60 pus cells/HPF), and sterile urine culture. Magnetic resonance imaging of the brain showed only age‐related atrophy (Figure 1). The patient was started on intravenous fluids and broad‐spectrum intravenous antibiotics at doses that were modified according to renal function. On the third day, her sensorium returned to normalcy and chorea resolved with improvement in her kidney function (urea: 82 mg/dL; creatinine: 2.0 mg/dL). She was diagnosed as having acute on chronic kidney disease with urinary tract infection and delirium, chorea secondary to uremia with background hypertension, and diabetes mellitus. The patient was discharged in a stable condition and she remained normal at the 6‐month follow‐up.

## 3 DISCUSSION

Patients with CKD can present with various nervous system manifestations, such as uremic encephalopathy, cerebrovascular accidents, cognitive dysfunction, and peripheral and autonomic neuropathies.^1^ Movement disorders appear rarely in uremic patients with symptoms of asterixis, myoclonus, and restless legs syndrome.^2^ Acute movement disorder manifesting as dyskinesias, akinetic, and parkinsonian syndromes has been described in diabetic uremic patients who were found to have bilateral basal ganglia lesions.^3^

Movement disorders can be defined as neurologic syndromes in which there is either an excess of movements (hyperkinesia) or a paucity of voluntary movements (dyskinesia) unrelated to weakness or spasticity. Chorea is a type of dyskinesia consisting of irregular movements that are involuntary, continual, abrupt, rapid, brief, non‐sustained and that flow from one body part to another. It may be the expression of a wide range of disorders (including metabolic, inflammatory, vascular, and drug‐induced conditions), which is classified as secondary chorea. Chorea is classified as primary when it is idiopathic or genetic in origin.^4^ The pathophysiology of chorea is poorly understood, but in contrast to parkinsonism and other movement disorders, intracortical inhibition of the motor cortex is normal in chorea. The semiquantitative analysis of single photon emission computed tomography in patients with hemichorea suggests that there is an increase in activity in the contralateral thalamus, possibly due to disinhibition as a result of the loss of normal pallidal inhibitory input.^5^ In CKD patients, uremic metabolites, dyselectrolytemia, and so forth can cause bilateral loss of normal pallidal inhibitory input, resulting in chorea.

In elderly patients presenting with acute‐ or subacute‐onset chorea, the following (reversible and irreversible) causes should be considered^6^: structural basal ganglia lesions, such as vascular chorea in stroke and extrapontine myelinolysis; infectious chorea seen in HIV encephalopathy, toxoplasmosis, cysticercosis, and bacterial endocarditis; post‐vaccinal encephalitis; paraneoplastic chorea; vasculitis‐like systemic lupus erythematosus; metabolic encephalopathies, which include hypo‐/hypernatremia, hypocalcaemia, hyperthyroidism, hypoparathyroidism, hepatic failure, and renal failure; and drug‐induced chorea, which includes dopamine receptor blocking agents, antiparkinsonian drugs, and antiepileptic drugs. Workup should be directed to find the underlying cause.

Chorea has been treated with drugs that interfere with central dopaminergic function, such as the dopamine receptor blocking drugs, reserpine and tetrabenazine. These medications may lessen patients' disabilities. Tetrabenazine provides the most effective relief of chorea with only minimal, dose‐related side‐effects, such as drowsiness, insomnia, depression, and parkinsonism.

There are two case reports of patients with CKD presenting with acute‐onset chorea.^7^, ^8^ Both patients were known to have diabetes with end‐stage renal disease and were undergoing hemodialysis. An increase in the frequency of hemodialysis over a few weeks resulted in symptom improvement.

In our case, chorea was due to a metabolic cause—uremia—that was treated with proper fluid management and treatment of underlying infection. In older adults with atypical symptoms, a thorough search for reversible causes and drug‐related adverse effects will help in the management and restoration of functionality.

## CONFLICT OF INTEREST

None of the authors has any conflict of interest to disclose.
